# Increasing HPV Vaccination Among Early Adolescents Using a Game-Based Digital Intervention: A Randomized Controlled Trial

**DOI:** 10.3390/vaccines14050448

**Published:** 2026-05-18

**Authors:** Angela Chia-Chen Chen, Lihong Ou, Elizabeth Reifsnider, Kimberly Arcoleo, Ashish Amresh, Michael Todd

**Affiliations:** 1 College of Nursing, Michigan State University, 1355 Bogue St, East Lansing, MI 48824, USA; arcoleok@msu.edu; 2 Edson College of Nursing and Health Innovation, Arizona State University, 550 N 3rd St, Phoenix, AZ 85004, USA; lihongou@asu.edu (L.O.); mike.todd@asu.edu (M.T.); 3 Ellmer School of Nursing, Old Dominion University, 1881 University Drive, Virginia Beach, VA 23453, USA; ereifsni@odu.edu; 4 School of Informatics, Computing, and Cyber Systems, Northern Arizona University, 1295 S Knoles Dr, Flagstaff, AZ 86011, USA; ashish.amresh@nau.edu

**Keywords:** HPV vaccination, adolescents, parents, serious game, digital intervention, randomized controlled trial

## Abstract

Background/Objectives: Human papillomavirus (HPV) vaccination coverage among adolescents remains below public health targets despite strong evidence of vaccine effectiveness in preventing HPV-related cancers. Digital interventions (e.g., serious games) may improve HPV vaccine uptake, but evidence for effects on vaccination behavior remains limited. Methods: This secondary analysis of a randomized controlled trial evaluated a co-designed, game-based digital intervention to increase HPV vaccine initiation among unvaccinated youth aged 11–14 years and their parents. The sample included 64 parent–adolescent dyads (33 intervention and 31 usual care dyads). The primary outcome was HPV vaccine initiation at 2-month follow-up. Results: A significantly greater proportion of adolescents in the intervention group initiated HPV vaccination compared with controls (88.5% vs. 46.2%; χ^2^(1) = 10.58, *p* = 0.001; risk difference = 0.423, 95% CI = [0.196, 0.650]). No significant between-group baseline differences were observed in parent HPV vaccination intention, knowledge, or psychosocial perceptions, although adolescent vaccination intention was higher in the intervention group. In adjusted logistic regression controlling for adolescent baseline HPV vaccination intention, intervention participants remained significantly more likely to initiate vaccination than controls (OR = 9.31, 95% CI = 2.13–40.70, *p* = 0.003). Intervention acceptability was high, with most parents and adolescents reporting that the game was easy to use, engaging, and relevant to vaccination decision-making. Conclusions: These findings provide preliminary evidence that a brief, family-centered, game-based digital intervention may help increase HPV vaccination initiation among adolescents. Larger trials with longer follow-up are needed to assess vaccine series completion and effectiveness across diverse settings.

## 1. Introduction

Human papillomavirus (HPV), the most common sexually transmitted infection in the United States and globally, is a leading cause of cervical, oropharyngeal, and other anogenital cancers affecting both females and males. According to the Centers for Disease Control and Prevention (CDC) [1], approximately 14 million new HPV infections occur annually, and HPV is responsible for about 37,000 cancer cases each year in the United States. HPV-associated diseases impose a substantial economic burden in the United States; updated national estimates indicate that the annual direct medical cost of HPV-related screening, follow-up, and treatment is approximately $9.01 billion in 2020 U.S. dollars [2].

The Advisory Committee on Immunization Practices (ACIP) recommends routine HPV vaccination beginning at ages 11 or 12, with catch-up vaccination through age 26 [3]. Despite strong national recommendations and well-documented vaccine effectiveness, HPV vaccination coverage among U.S. adolescents remains below the Healthy People 2030 target of 80% series completion [4]. Recent CDC data indicate that approximately 62% of U.S. adolescents are up to date with the HPV vaccine series [5,6,7,8]. Although coverage has improved over time, important differences persist across populations and geographic settings. These gaps underscore the need for innovative, developmentally appropriate, and family-centered strategies that effectively engage families and translate HPV vaccine awareness into timely vaccination initiation.

Multiple factors contribute to suboptimal HPV vaccination uptake in the United States. Barriers include limited knowledge and awareness, misconceptions about vaccine safety, concerns about sexual stigma, inconsistent provider recommendations, and structural barriers such as limited access to pediatric care and transportation challenges [8,9,10]. Recent evidence further highlights missed opportunities for timely vaccination, showing that approximately 12% of adolescents initiate sexual activity before receiving HPV vaccination, with clinician practices and clinic workflow identified as key contributors to delayed uptake [11]. Although HPV vaccination decisions for early adolescents are largely determined by caregivers, adolescents’ perceptions, engagement with health information, and communication within the family context may shape parental decision-making. This developmental context highlights the importance of interventions that engage both adolescents and caregivers. Traditional health education approaches that rely primarily on printed materials or brief clinical counseling may be insufficient to address these complex psychosocial and contextual determinants and may not sustain adolescents’ engagement with preventive health information [12,13].

In response to these challenges, interactive digital interventions, particularly serious games designed for educational and behavioral purposes, have emerged as promising tools to enhance engagement, facilitate knowledge acquisition, and support motivation for preventive health behaviors [14,15]. Prior studies have shown that brief digital interventions, including computer-based modules, mobile applications, serious games, and animated educational videos, can improve HPV-related knowledge and increase vaccination intention among adolescents and parents [16,17]. These findings suggest that interactive digital approaches may help translate awareness into vaccination-related decision-making and behavior.

Recent systematic evidence indicates that while game-based vaccination interventions improve knowledge and engagement, robust evidence demonstrating effects on actual vaccination uptake remains limited. A synthesis of existing studies identified relatively few randomized trials and highlighted heterogeneity in intervention design and behavioral outcome assessment, underscoring the need for rigorously designed trials evaluating real-world vaccination behavior [18].

Building on this evidence, this study used a randomized controlled trial to evaluate HPV Detective, a co-designed, game-based digital intervention developed with input from youth and parents to promote HPV vaccination for cancer prevention among adolescents through immersive and interactive learning [19]. The primary aim was to examine the preliminary efficacy of the intervention in increasing HPV vaccine initiation by comparing vaccination uptake between parent-adolescent dyads assigned to the game intervention and those receiving usual care. By testing a family-centered serious game in a randomized controlled trial, this study provides preliminary efficacy evidence for an approach that engages adolescents as primary users while involving parents and caregivers in vaccination decision-making. The findings highlight the potential advantage of interactive, family-centered digital strategies in translating engagement into preventive health behaviors and may inform the development of scalable approaches to improve vaccination coverage and reduce HPV-related cancer burden among adolescents.

## 2. Materials and Methods

### 2.1. Study Design

The data for this study were derived from a randomized controlled trial evaluating two game-based vaccination interventions (HPV and COVID-19) and a usual care control condition among adolescents and their parents; the COVID-19 versus usual care comparison has been previously published [17]. The trial used consistent eligibility criteria, recruitment strategies, and procedures across study arms, with measures tailored to the specific vaccine context. The present secondary analysis focuses on participants randomized to the HPV intervention and usual care control groups. The parent trial enrolled and randomized 96 parent–adolescent dyads across three study arms. The present secondary analysis included 64 dyads randomized to the HPV intervention (*n* = 33) or usual care (*n* = 31). As a pilot trial, the study was intended to generate preliminary efficacy and feasibility data rather than provide definitive hypothesis testing based on a formal power calculation.

### 2.2. Participants and Recruitment

Recruitment materials, including research flyers and brief promotional videos, were disseminated through the university’s internal newsletter, local immunization and nursing organizations, community libraries, and social media platforms (e.g., Facebook and Twitter). Additional participants were identified through snowball sampling and word-of-mouth referrals. Interested individuals accessed a QR code on the flyers to complete an online eligibility screening survey, followed by a brief one-on-one Zoom screening. All surveys were self-administered using Research Electronic Data Capture (REDCap) [20].

Eligibility criteria included youth aged 11–14 years who had not initiated HPV vaccination and one parent or legal guardian able to provide informed consent. Exclusion criteria included prior receipt of any HPV vaccine dose or inability to complete study procedures in English.

Eligible participants who completed the pretest survey were randomized into either the intervention or usual care groups through REDCap’s randomization module using simple randomization. Allocation was concealed until completion of the baseline assessment and randomization process. Dyads in the intervention group received the game-based HPV Detective intervention, while those in the usual-care group continued their usual activities and did not receive intervention-related content during the study period. Blinding of participants and study staff was not feasible due to the behavioral nature of the intervention; however, outcome analyses were conducted by team members blinded to group allocation.

### 2.3. Intervention

The HPV Detective game was developed using a participatory design approach with input from key stakeholders, including youth and parents, to provide HPV-related education and support vaccination decision-making among adolescents and their caregivers. Theoretical guidance was drawn from Health Belief Model constructs, including perceived risk/susceptibility, perceived benefits, perceived barriers, and cues to action, with vaccination communication incorporated as a key mechanism to prompt preventive behavior [18,21]. The intervention incorporates interactive gameplay elements such as knowledge challenges, narrative-based missions, and point-based rewards to enhance engagement and learning. Adolescents served as the primary users of the game, while caregivers were encouraged to participate alongside them to facilitate shared understanding and communication regarding HPV vaccination. The intervention was designed to be completed in a single session in approximately 10–15 min and was delivered digitally [17,22].

### 2.4. Data Collection

After providing informed consent and assent, parent–youth dyads completed a baseline assessment (T0) and were randomly assigned to either the game-based intervention group or the usual-care control group. The intervention group completed additional assessments immediately after gameplay (T1) and at a 2-month follow-up (T2), while the usual care group completed assessments at baseline and T2. Participants received text reminders before the 2-month follow-up survey to encourage completion. Each participant received a $10 incentive for each completed survey as compensation for their time.

### 2.5. Measures

All study measures were adapted from instruments used in our prior research with parents and adolescents and have demonstrated acceptable psychometric properties [19,22].

#### 2.5.1. Primary Outcome: HPV Vaccine Initiation

The primary outcome was initiation of the human papillomavirus (HPV) vaccine, defined as receipt of at least one dose of the HPV vaccine by the 2-month follow-up (T2). Vaccination status was reported by parents at follow-up.

#### 2.5.2. HPV Vaccination Intention

Parent and adolescent HPV vaccination intentions were assessed at baseline and follow-up using a single-item measure with three response options (“no,” “maybe,” and “yes”), with higher responses indicating stronger vaccination intention. Single-item intention measures were used to reduce participant burden and were considered appropriate for assessing a focused behavioral intention in this pilot study. This approach was also consistent with measures used in our prior HPV vaccination research. Parents’ intention to vaccinate their adolescent was assessed at baseline (T0), immediately post-intervention (T1; intervention group only), and at 2-month follow-up (T2). Adolescent’s own vaccination intention was assessed at baseline (T0) and immediately post-intervention (T1; intervention group only) only, in order to minimize adolescent participant burden.

#### 2.5.3. HPV Knowledge

Parent and adolescent HPV knowledge were assessed at baseline using 10-item measures covering HPV transmission, consequences, and vaccine-related information. Responses were recorded as correct (1) or incorrect (0), and summed to generate individual total knowledge scores, with higher scores indicating greater HPV knowledge. In this sample, the parent and adolescent HPV knowledge scale demonstrated acceptable internal consistency (Cronbach’s α = 0.70 and 0.75, respectively).

#### 2.5.4. Psychosocial Measures

Parents’ psychosocial perceptions related to HPV vaccination were assessed at baseline using constructs informed by the Health Belief Model, including perceived adolescent HPV risk, perceived facilitators of HPV vaccination, and perceived barriers to vaccination.

Perceived adolescent HPV risk was assessed using 8 items reflecting beliefs that the adolescent was at low risk for HPV infection. Items were coded 0 = no and 1 = yes, summed, and scored such that higher scores indicated lower perceived risk. Internal consistency in this sample was acceptable (Cronbach’s α = 0.76).

Perceived facilitators were assessed using 9 items representing conditions that would encourage HPV vaccination, such as provider recommendation, social encouragement, affordability, and knowledge of where to obtain the vaccine. Items were coded dichotomously (0 = no and 1 = yes) and summed, with higher scores indicating more perceived facilitators. The scale demonstrated good internal consistency (Cronbach’s α = 0.84).

Perceived barriers were assessed using 21 items examining concerns and obstacles related to HPV vaccination, including beliefs about safety, stigma, cost, access, and uncertainty about vaccine effectiveness. Items were coded dichotomously (0 = no and 1 = yes) and summed, with higher scores indicating more perceived barriers. Internal consistency was good (Cronbach’s α = 0.86).

#### 2.5.5. Intervention Acceptability and Gaming Experience

Intervention acceptability and gaming experience were assessed among participants in the intervention group immediately after gameplay (T1). The measure consisted of 15 items evaluating usability (e.g., ease of following the game and understanding instructions), engagement (e.g., attention holding), perceived usefulness (e.g., relevance of information and support for vaccination decision-making), and overall satisfaction (e.g., recommendation and game length). Items were rated on ordinal response scales by item (e.g., “very difficult” to “very easy,” “not at all” to “a lot,” or agreement scales). Higher scores reflected greater perceived acceptability and more positive gaming experiences.

### 2.6. Statistical Analysis

For this secondary analysis, statistical comparisons were limited to participants randomized to the HPV intervention group and the usual care control group. Quantitative data were analyzed using IBM SPSS Statistics version 31.0 (IBM Corp., Armonk, NY, USA) [23]. Descriptive statistics were used to summarize participant demographics and baseline characteristics. Between-group differences were examined using chi-square and Fisher’s exact tests. To evaluate intervention effects on HPV vaccination initiation, a chi-square test was conducted to compare vaccination proportions between groups at follow-up. The effect size was estimated as the absolute risk difference. Because adolescent HPV vaccination intention differed between groups at baseline, an additional binary logistic regression analysis was conducted to examine whether group assignment remained associated with adolescent’s HPV vaccine initiation after adjusting for adolescent baseline vaccination intention. Group assignment and adolescent baseline intention were entered simultaneously as predictors, and adjusted odds ratios (ORs) with 95% confidence intervals (CIs) were reported. Statistical significance was set at *p* < 0.05 (two-tailed).

### 2.7. Ethical Approvals

This study was approved by the Arizona State University Institutional Review Board (Approval Number: STUDY00016412). Written informed consent and assent were obtained from all participants prior to enrollment, and participation was entirely voluntary.

## 3. Results

### 3.1. Participant Characteristics

**Figure 1:**
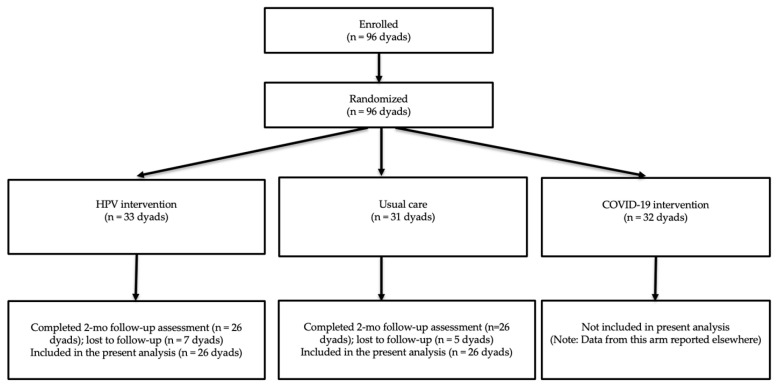
CONSORT-style flow diagram of participant progression through the parent randomized controlled trial and participants included in the present secondary analysis. The parent trial included three study arms (HPV intervention, COVID-19 intervention, and usual care). The present secondary analysis focuses on participants in the HPV intervention and usual care groups with available 2-month follow-up vaccination outcome data.

Participant flow through the parent trial and the subset included in the present secondary analysis is shown in Figure 1. The parent trial included three study arms (HPV intervention, COVID-19 intervention, and usual care), whereas the present analysis focused on the HPV intervention and usual care groups. For the present secondary analysis, 64 parent–adolescent dyads from the parent trial were randomized to the HPV intervention (*n* = 33) or usual care group (*n* = 31). Among these 64 dyads, 52 completed the 2-month follow-up (T2) vaccination outcome assessment, resulting in an overall attrition rate of 18.8% (12/64). Attrition was 21.2% in the intervention group (7/33) and 16.1% in the usual care group (5/31). Reasons for attrition were not available because participants who did not complete the T2 survey did not provide follow-up information. Baseline demographic characteristics of parents and adolescents were comparable between groups, with no statistically significant between-group differences observed.

### 3.2. Baseline Knowledge and Psychosocial Measures

At baseline (T0), parents’ HPV-related knowledge and psychosocial constructs were assessed. No statistically significant between-group differences were observed in parent HPV knowledge, perceived adolescent infection risk, perceived facilitators, perceived barriers, or adolescent’s HPV knowledge. Detailed baseline statistics are presented in Table 1.

### 3.3. Baseline HPV Vaccination Intention

At baseline (T0), parent HPV vaccination intention did not differ significantly between groups (χ^2^(2) = 4.80, *p* = 0.09). However, adolescent HPV vaccination intention differed between groups, with a greater proportion of adolescents in the intervention group reporting intention to receive the HPV vaccine (χ^2^(2) = 6.31, *p* = 0.04).

### 3.4. HPV Vaccine Initiation at Follow-Up

At the 2-month follow-up (T2), a significantly greater proportion of adolescents in the intervention group had initiated HPV vaccination compared with those in the usual care group (23/26 [88.5%] vs. 12/26 [46.2%]; χ^2^(1) = 10.58, *p* = 0.001). The absolute risk difference in vaccination initiation between groups was 42.3 percentage points (risk difference = 0.423, 95% CI [0.196, 0.650]). This corresponds to approximately 42 additional adolescents initiating HPV vaccination for every 100 adolescents exposed to the intervention compared with usual care. In an additional binary logistic regression analysis controlling for adolescent baseline HPV vaccination intention, adolescents in the intervention group remained significantly more likely to initiate HPV vaccination than those in the usual care group (OR = 9.31, 95% CI = 2.13–40.70, *p* = 0.003). Adolescent baseline vaccination intention was not a significant independent predictor of vaccine initiation (*p* = 0.789).

### 3.5. Intervention Acceptability and Gaming Experience

Overall, parents and adolescents reported positive gaming experiences. Most participants indicated that the game was easy to follow and understand, with approximately 80–90% reporting that the format and instructions were somewhat or very easy. The majority also reported that the information was easy to understand (parents: 92%; adolescents: 96%) and that the game held their attention (parents: 92%; adolescents: 93%). More than 70% of both parents and adolescents reported that the information was relevant to their lives and addressed their informational needs. Nearly half of parents (48%) and more than half of adolescents (59%) indicated that the game strongly supported their HPV vaccination decision-making. In addition, most participants agreed or strongly agreed that the game was a good way to learn about HPV vaccination and that they would recommend it to others. The majority perceived the game length as adequate (parents: 70%; adolescents: 82%). Detailed distributions are presented in Table 2. No intervention-related adverse events were reported.

## 4. Discussion

The findings of this study provide preliminary evidence that a brief, game-based digital intervention may increase HPV vaccination initiation among early adolescents. Adolescents exposed to the intervention were substantially more likely to initiate HPV vaccination within two months compared with those receiving usual care. Consistent with prior research, our findings show that digital interventions can improve HPV-related knowledge and vaccination intention among adolescents and parents [16]. While many earlier studies reported improvements primarily in psychosocial outcomes such as knowledge, attitudes, or intention [14,17], the present study extends the literature by suggesting a potentially meaningful effect on actual vaccination behavior. This aligns with emerging evidence suggesting that interactive digital formats may be particularly effective for engaging adolescents and facilitating health behavior changes when interventions are theory-driven and developmentally tailored [13,15,24].

The findings are also consistent with broader literature emphasizing the importance of family-centered approaches in HPV vaccination promotion. Prior studies have shown that caregiver beliefs, communication patterns, and perceived barriers play critical roles in vaccination decision-making [9,10]. By engaging both adolescents and parents, the intervention may have facilitated family communication and increased the perceived importance of HPV vaccination. Although the intervention was designed to encourage parent–adolescent communication, actual communication processes and the degree of adolescent influence on vaccination decisions were not directly assessed. This family-dyad approach aligns with developmental frameworks highlighting shared health decision-making during early adolescence and may represent a key mechanism through which digital interventions influence preventive health behaviors.

High levels of acceptability and engagement observed in this study further support the feasibility of implementing game-based interventions for HPV vaccination promotion. Most parents and adolescents reported that the game was easy to use, engaging, and relevant, with a majority indicating that it held their attention, addressed their informational needs, and supported vaccination decision-making. These findings are consistent with prior evaluations of serious games in vaccination and adolescent health promotion, which have demonstrated strong engagement and positive user experiences [14,17]. Engagement is a critical determinant of digital intervention effectiveness, particularly among adolescents who may be less responsive to traditional educational approaches. The brief duration and positive user experience reported in this study suggest that such interventions may be feasibly integrated into clinical, school-based, or community vaccination programs.

Baseline findings indicated no significant between-group differences in parent vaccination intention, knowledge, or psychosocial perceptions; however, a higher proportion of adolescents in the intervention group reported vaccination intention at baseline. To address this baseline imbalance, an additional adjusted logistic regression analysis demonstrated that adolescents in the intervention group remained significantly more likely to initiate HPV vaccination after controlling for baseline adolescent vaccination intention. This finding suggests that the observed intervention effect was not explained by baseline differences in adolescent vaccination intention, which was not an independent predictor of vaccine initiation. Future trials with larger samples should consider stratified randomization or statistical adjustment for baseline motivational differences. Additionally, theory-driven analyses using structural equation modeling could help clarify the pathways through which the intervention influences vaccination behavior.

Several limitations should be considered. The relatively small sample size and short follow-up period limit generalizability and preclude assessment of long-term vaccination completion. Given the pilot sample size, effect estimates should be interpreted cautiously, as the observed effect sizes may be imprecise until confirmed in larger adequately powered trials. Vaccination status was parent-reported and not independently verified through medical records, immunization registries, or provider documentation, which may introduce reporting bias, recall error, or social desirability bias. As a result, misclassification of vaccination status is possible. Additionally, the sample had relatively high educational attainment and was recruited primarily through community-based and online strategies, which may limit generalizability to families with lower educational attainment, limited digital access, or different clinical/community contexts. Although adolescent HPV vaccination intention was higher in the intervention group at baseline, additional adjusted analyses controlling for baseline intention yielded similar findings, suggesting that the observed intervention effect was not explained by this difference. In addition, while the 2-month follow-up period was appropriate for assessing vaccine initiation, it did not permit evaluation of HPV vaccine series completion or longer-term sustainability of intervention effects. These limitations are consistent with challenges observed in early-stage trials of digital vaccination interventions [16]. Future research should include larger and more diverse populations, longer follow-up periods, and objective verification of vaccination outcomes.

Despite these limitations, this study contributes to the growing evidence base supporting digital interventions for HPV vaccination promotion by demonstrating that a co-designed serious game can influence vaccination behavior. Overall, the findings suggest that theory-informed, family-centered digital interventions may represent a promising and scalable strategy for improving HPV vaccination uptake among adolescents. Such approaches may help translate HPV vaccine awareness and intention into actual vaccination behavior through engaging and interactive learning experiences. Continued evaluation through larger randomized trials will be important to determine scalability and long-term impact on HPV vaccine completion.

## 5. Conclusions

A game-based digital intervention was associated with increased HPV vaccination initiation among early adolescents in this randomized trial. The intervention was well accepted, engaging, and feasible for delivery to parent–adolescent dyads. Given that vaccination behaviors are context-dependent, further research is needed to evaluate long-term vaccine series completion, effectiveness in larger and more diverse populations, and implementation in real-world clinical and community settings. Potential implementation settings may include pediatric clinics, school-based health programs, community organizations, and public health outreach initiatives serving families with adolescents. As a brief digital intervention requiring minimal personnel time, this approach may offer a potentially scalable and cost-efficient strategy, although a formal economic evaluation is needed. Future trials should further address baseline group imbalances through stratified randomization or prespecified adjusted analyses and examine long-term vaccine series completion, dissemination, and sustainability. If demonstrated to be effective in larger trials, such scalable digital approaches may support efforts to improve HPV vaccination uptake and reduce HPV-related cancer burden at the population level.

## Figures and Tables

**Table 1:** Baseline characteristics of parent–adolescent dyads (N = 64 dyads).

Characteristic	Intervention (*n* = 33)	Usual Care (*n* = 31)	Test Statistic (t/χ2)	*p*-Value
Parent Characteristics				
Age (mean ± SD)	36.0 (5.8)	33.7 (4.7)	1.71	0.09
Male (*n*, %)	22 (66.7%)	20 (66.7%)	0.00	1.00
Black race (*n*, %)	24 (72.7%)	26 (86.7%)	4.95	0.18
Employed (*n*, %)	28 (84.8%)	27 (90.0%)	0.38	0.54
Level of education (n, %)				
Some high school	0 (0.0%)	1 (3.3%)	4.41	0.35
Finished high school/GED	2 (6.1%)	1 (3.3%)		
Some vocational or college training	2 (6.1%)	2 (6.7%)		
Finished bachelors degree	20 (60.6%)	23 (76.7%)		
Finished graduate degree	9 (27.3%)	3 (10.0%)		
Adolescent Characteristics				
Age (mean ± SD)	12.36 (1.1)	12.42 (0.8)	−0.24	0.81
Male (n, %)	25 (75.8%)	23 (74.2%)	0.02	0.89
Free/reduced-price lunch (n, %)	16 (48.5%)	13 (43.3%)	0.17	0.68
Theoretical Variables				
Parent HPV Knowledge (mean ± SD)	8.73 (1.42)	7.97 (2.24)	1.59	0.12
Perceived Adolescent HPV Risk (mean ± SD)	12.79 (2.18)	12.16 (2.13)	1.16	0.25
Perceived Facilitators (mean ± SD)	4.97 (2.73)	4.61 (2.76)	0.52	0.61
Perceived Barriers (mean ± SD)	5.94 (5.05)	6.39 (4.31)	−0.38	0.71
Adolescent HPV Knowledge (mean ± SD)	8.76 (1.68)	8.55 (2.05)	0.44	0.67
Parent Vaccination Intention (*n*, %)				
Yes	16 (48.5%)	8 (25.8%)	4.80	0.09
Maybe	9 (27.3%)	8 (25.8%)		
No	8 (24.2%)	15 (48.4%)		
Adolescent Vaccination Intention (*n*, %)				
Yes	16 (48.5%)	7 (23.3%)	6.31	0.04
Maybe	9 (27.3%)	7 (23.3%)		
No	8 (24.2%)	16 (53.3%)		

Abbreviations: SD = Standard deviation; GED = General Educational Development; HPV = Human Papillomavirus.

**Table 2:** Intervention acceptability and gaming experience among parent–adolescent dyads in the intervention group (N = 27 dyads).

Ratings of Gaming Experience	Parent (*n*, %)	Adolescent (*n*, %)
Game Playing		
Game format easy to follow		
(Somewhat difficult)	5 (18.5)	2 (7.4)
(Somewhat easy)	13 (48.1)	13 (48.1)
(Very easy)	9 (33.3)	12 (44.4)
Game instruction easy to understand		
(Somewhat difficult)	4 (14.8)	4 (14.8)
(Somewhat easy)	11 (40.7)	9 (33.3)
(Very easy)	12 (44.4)	14 (51.9)
Words in the game are easy to see		
(Very difficult)	0 (0.00)	1 (3.7)
(Somewhat difficult)	2 (7.4)	1 (3.7)
(Somewhat easy)	8 (29.6)	9 (33.3)
(Very easy)	17 (63.0)	16 (59.3)
Information in game easy to understand		
(Somewhat difficult)	2 (7.7)	1 (3.7)
(Somewhat easy)	8 (30.8)	8 (29.6)
(Very easy)	16 (61.5)	18 (66.7)
Game Like		
Graphics and design		
(Not at all)	1 (3.7)	1 (3.7)
(A little)	6 (22.2)	3 (11.1)
(Somewhat)	6 (22.2)	4 (14.8)
(A lot)	14 (51.9)	19 (70.4)
Attention hold		
(Not at all)	0 (0.0)	2 (7.4)
(A little)	2 (7.4)	0 (0.0)
(Somewhat)	9 (33.3)	4 (14.8)
(A lot)	16 (59.3)	21 (77.8)
Information relevant to life		
(Not at all)	0 (0.0)	1 (3.7)
(A little)	4 (14.8)	3 (11.1)
(Somewhat)	4 (14.8)	3 (11.1)
(A lot)	19 (70.4)	20 (74.1)
Informational needs addressed		
(Not at all)	1 (3.7)	1 (3.7)
(A little)	4 (14.8)	1 (3.7)
(Somewhat)	3(11.1)	5 (18.5)
(A lot)	19 (70.4)	20 (74.1)
Decision support in HPV vaccination		
(Not at all)	1 (3.7)	3 (11.1)
(A little)	3 (11.1)	3 (11.1)
(Somewhat)	10 (37.0)	5 (18.5)
(A lot)	13 (48.1)	16 (59.3)
Good way to learn about the HPV vaccine		
(Disagree)	2 (7.4)	1 (3.7)
(Agree)	13 (48.1)	17 (63.0)
(Strongly agree)	12 (44.4)	9 (33.3)
Game Recommendation		
(Disagree)	3 (11.1)	2 (7.4)
(Agree)	11(40.7)	11(40.7)
(Strongly agree)	13 (48.1)	14 (51.9)
Game Length		
(Way too long)	1 (3.7)	1 (3.7)
(Somewhat long)	6 (22.2)	4 (14.8)
(Adequate)	19 (70.4)	22 (81.5)
(Short)	1 (3.7)	0 (0.0)

Note: N = 27 dyads reflect immediate post-intervention respondents, not the T2 follow-up sample.

## Data Availability

The data supporting the findings of this study are available from the corresponding author upon reasonable request and with appropriate Institutional Review Board approval.
